# Test–retest reliability and sensitivity of horizontal jump inter-limb asymmetry in youth soccer players across maturity stages

**DOI:** 10.1371/journal.pone.0341344

**Published:** 2026-06-22

**Authors:** Raouf Hammami, Yassine Negra, Halil Ibrahim Ceylan, Walid Selmi, Ryland Morgans, Francisco Tomás González-Fernández, Nicola Luigi Bragazzi

**Affiliations:** 1 University of Manouba, Higher Institute of Sport and Physical Education of Ksar-Said, University Campus, Manouba, Tunisia; 2 Tunisian Research Laboratory ‘Sports Performance Optimization’, National Center of Medicine and Science in Sports (CNMSS-LR09SEP01), Tunis, Tunisia; 3 Research Laboratory (LR23JS01) «Sport Performance, Health & Society», Higher Institute of Sport and Physical Education of Ksar Saïd, University of La Manouba, La Manouba, Tunisia; 4 Physical Education and Sports Teaching Department, Faculty of Sports Sciences, Ataturk University, Erzurum, Turkey; 5 School of Sport and Health Sciences, Cardiff Metropolitan University, Cardiff, United Kingdom; 6 Department of Physical Education and Sports, Faculty of Sport Sciences, University of Granada, Granada, Spain; 7 Laboratory for Industrial and Applied Mathematics (LIAM), Department of Mathematics and Statistics, York University, Toronto, Canada; 8 Human Nutrition Unit (HNU), Department of Food and Drugs, University of Parma, Medical School, Parma, Italy; University of Urbino: Universita degli Studi di Urbino Carlo Bo, ITALY

## Abstract

The present study examined the test–retest reliability and sensitivity of inter-limb asymmetry derived from the single-leg hop test (SLHT) across different maturity stages in young male soccer players. Sixty-four youth soccer players aged 8–16 years participated in this study and were assessed using the SLHT to quantify inter-limb asymmetry. Participants were divided into prepubertal (n = 34; age: 8.1 ± 1.4 years; body mass: 35.7 ± 3.4 kg; height: 122 ± 9 cm; maturity offset: −2.38 ± 0.83 years) and post-pubertal groups (n = 30; age: 16.5 ± 0.3 years; body mass: 39.6 ± 6.1 kg; height: 154 ± 7 cm; maturity offset: 1.17 ± 0.45 years) according to individual peak height velocity. During the SLHT, participants performed maximal horizontal hops on each leg separately, and the greatest distance achieved was recorded for analysis. Inter-limb asymmetry demonstrated good relative reliability in both groups, with intraclass correlation coefficients of 0.78 and 0.92 for the prepubertal and post-pubertal groups, respectively. Absolute reliability was also acceptable, as the typical error of measurement was < 5% in both groups. Sensitivity analysis showed that the standard error of measurement (SEM) exceeded the smallest worthwhile change (SWC; 0.2 standard deviations) in the prepubertal group, indicating a marginal ability to detect small performance changes. In contrast, the SEM was lower than the SWC in the post-pubertal group, indicating good sensitivity for detecting small changes in performance. These findings indicate that SLHT-derived inter-limb asymmetry scores demonstrate acceptable reliability in youth soccer players across maturity stages, while sensitivity to detect small changes may differ between prepubertal and post-pubertal players. The results contribute to the methodological understanding of inter-limb asymmetry assessment in youth soccer and may assist practitioners and researchers in interpreting asymmetry data across different stages of maturation.

## Background

Morphological differences among young soccer players have been widely documented across chronological age groups [1], with pronounced changes occurring during adolescence [2]. These differences can directly affect coordinative abilities and contribute to the development of inter-limb asymmetries, which should be identified and addressed in youth soccer players. Lower-limb asymmetries have been associated with reduced physical performance [3,4], and their emergence may be linked to the substantial morphological and neural changes that occur during growth and maturation [5]. In particular, the transitional period around peak height velocity (PHV) is often characterized by temporary losses in motor control, negatively influencing balance, strength, and jump [6,7].

Because maturation does not occur uniformly, significant inter-individual variability in the timing and rate of biological changes is observed even among athletes of the same chronological age [5]. This variability can contribute to large discrepancies in physical and physiological performance measures and partly explain decrements in neuromuscular control during adolescence [8]. Asymmetry in youth athletes is typically considered meaningful when bilateral differences exceed 10–15% [9,10], though the most appropriate assessment procedures remain debated. For example, unilateral jump tests have demonstrated high reliability in elite youth female soccer players (ICC = 0.72–0.99; CV = 2.7–5.7%) [11], but recent findings suggest that asymmetry should be interpreted individually and across multiple metrics, since the favored limb often differs depending on the test used [11]. This highlights the need for normative data and flexible reference thresholds for practitioners.

Performance differences related to maturity status have also been observed. Pardos-Mainer et al. [12] reported significant asymmetries in change-of-direction (COD) and jump tests among U-18, U-16, and U-14 female soccer players, with large effect sizes, although these asymmetries did not necessarily impair overall performance. Collectively, such findings suggest that inter-limb asymmetries are common during adolescence and may increase with maturation. However, the evidence remains inconclusive regarding their impact on performance and the extent to which they are influenced by chronological versus biological age.

Moreover, prepubertal players are limited in their adaptive responses compared to more mature peers, with neural mechanisms—such as enhanced excitation-contraction coupling—likely playing a predominant role in improvements in jumping [13–16]. This raises further questions about how maturation shapes both the presence and reliability of inter-limb asymmetry measures in youth soccer players.

Given these uncertainties, further research is warranted to better understand the measurement properties of inter-limb asymmetry assessments across different stages of maturation in young athletes. Therefore, the purpose of the present study was to investigate the test–retest reliability and sensitivity of inter-limb asymmetry across different maturity states in youth soccer players. Based on previous findings, we hypothesized that strength-based inter-limb asymmetry measures would demonstrate acceptable absolute and relative reliability regardless of maturity status.

## Methods

### Participants

Sixty-four male youth soccer players participated in this study. In line with the participant classification framework proposed by McKay et al. [17] and operationalised scoring criteria outlined by Wilkins et al. [18], the sample can be classified as recreationally trained youth athletes competing within a structured club-based development pathway. All participants were involved in organised soccer training and regular competition within the same regional youth league structure.

The sample was further stratified according to maturity status into a prepubertal group (n = 34; age: 8.1 ± 1.4 years; body mass: 35.7 ± 3.4 kg; height: 122 ± 9 cm; maturity offset: −2.38 ± 0.83 years) and a post-pubertal group (n = 30; age: 16.5 ± 0.3 years; body mass: 39.6 ± 6.1 kg; height: 154 ± 7 cm; maturity offset: 1.17 ± 0.45 years) (Table 1).

**Table 1:** Participant anthropometric characteristics (mean±standard deviation).

Characteristics	Pre-APHV (n = 34)	Post-APHV (n = 30)	
**Age (yrs)**	8.13 ± 1.40	16.51 ± 0.33	0.000
**Body mass (kg)**	35.74 ± 3.36	39.55 ± 6.09	0.000
**Standing height (cm)**	122 ± 9	154 ± 7	0.000
**Body fat**	10.20 ± 3.88	10.97 ± 3.48	0.403
**Maturity status (yrs)**	−2.38 ± 0.83	1.17 ± 0.45	0.000
**APHV (yrs)**	12.51 ± 0.59	15.34 ± 0.40	0.000

APHV = age at peak height velocity

The sample size was determined a priori using G*Power version 3.1.9.7 (University of Düsseldorf, Germany). The calculation was based on a mixed-model ANOVA (test family: F-tests; statistical test: repeated-measures, within–between interaction), with two groups (prepubertal vs. post-pubertal) and two measurement time points. A projected medium effect size (f = 0.25), α level of 0.05, statistical power of 0.80, and an assumed correlation among repeated measures of 0.50 were used. These parameters were derived from previous youth soccer and maturity-related research. The analysis indicated that a minimum of 52 participants was required; therefore, 64 players were recruited to account for potential attrition.

Goalkeepers were excluded due to the distinct physical and technical demands of the position and their lower involvement in high-frequency running actions. Outfield players were classified into playing positions following established methodologies: center backs (n = 9), full-backs (n = 6), central midfielders (n = 12), attacking midfielders (n = 10), and center forwards (n = 7).

All participants had at least 4–5 years of structured soccer training experience, comprising five 90-minute training sessions per week and one competitive match at the weekend. Inclusion criteria required full attendance across all testing sessions, absence of injury throughout the study period, and no use of dietary supplements or participation in additional structured training outside their club programme. Only players with 100% adherence to testing procedures were included in the final analysis.

All participants and their legal guardians were informed about the study procedures, potential risks, and benefits, and written informed consent was obtained prior to participation. Players were informed of their right to withdraw at any stage. Data were anonymised prior to analysis. The study was conducted in accordance with the Declaration of Helsinki and approved by the Ethics Committee for the Protection of Southern People, Tunisia Ministry of Health (decision no: CPP SUD0219, date: 02/08/2023), as well as the participating professional club.

### Procedures

All field tests were conducted between September 1 and 10, 2023, on a third-generation synthetic soccer pitch, at a consistent time of day (17:00–19:00 h), and under stable environmental conditions (temperature: 19–23 °C; relative humidity: 50–60%; wind speed: ≤ 2 m·s ⁻ ¹). Players were instructed to give maximal effort throughout all assessments. To minimize potential learning effects, two orientation sessions were held one week before the start of the study, allowing participants to become familiar with the testing environment, equipment, and procedures. Both familiarization and testing were supervised by the same strength and conditioning coaches.

Before testing, participants completed a standardized 15-minute warm-up consisting of submaximal running, dynamic stretching, and progressive plyometric exercises. Testing took place across two separate days, separated by 48 hours of rest. The study was carried out during the first half of the competitive season (November 2023), with all players following similar training and match schedules during the testing week.

**Fig 1:**
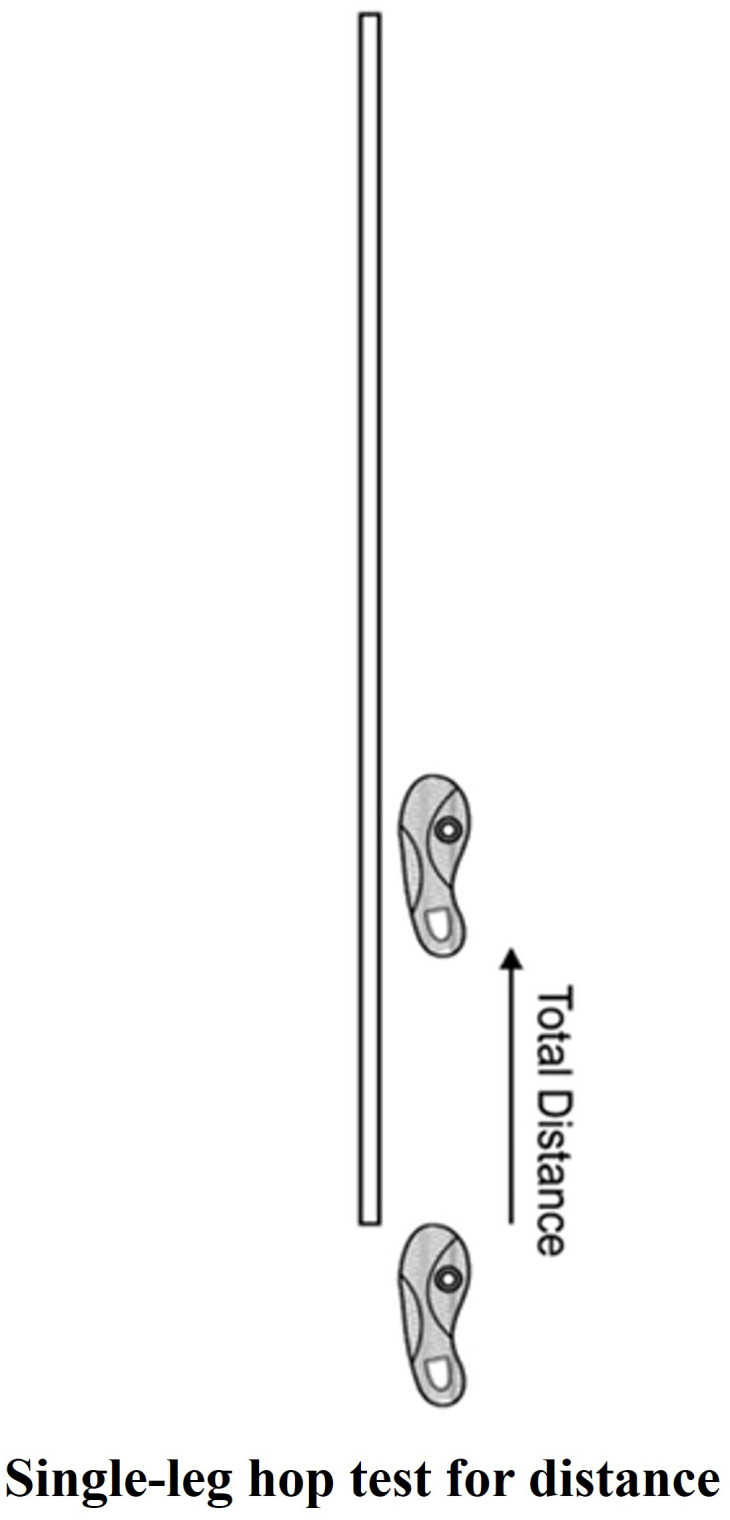
Schematic representation of the single-leg hop test for distance.

Following the warm-up, participants performed the single-leg hop for distance test, a widely used measure of functional performance in youth soccer players [19]. Each trial began with the athlete standing still on the designated test leg, with hands placed on the hips and toes positioned just behind the starting line. Standardized verbal instructions were provided to all participants: they were instructed to “jump as far forward as possible using one leg and stick the landing while maintaining balance for approximately 2 seconds” (Fig 1). To standardize propulsion strategy across participants, hands remained fixed on the hips throughout the movement, while a natural swing-leg action was permitted during the hop to allow coordinated movement execution. Emphasis was placed on achieving maximal horizontal distance and stable landing performance. A trial was considered valid if the participant achieved maximal distance without losing balance upon landing. Each leg was tested twice, and the best performance was retained for analysis. The same coach who conducted the familiarization sessions also administered all tests to ensure consistency of instructions and testing procedures.

Maturation status was approximated utilizing a formula proposed by previous research [20], wherein maturity status was calculated as: maturity status = −7.999994 + (0.0036124 × age [years] × height [centimeters]). To determine the magnitude and direction of inter-limb asymmetry, the lower limb asymmetry formula modified by Bishop et al. [19] was used: ((100/(maximum value)) × (minimum value) × −1 + 100) × IF(left < right, 1, −1). This approach incorporates the Excel IF function, allowing the direction of asymmetry (left vs. right) to be identified without affecting the magnitude of the asymmetry score (expressed as %).

To ensure methodological transparency, testing of the dominant and non-dominant limbs was conducted in a fixed order that was consistent across all participants and both testing sessions, thereby maintaining standardisation of measurement conditions.

### Statistical analysis

Data was expressed as mean and standard deviation (SD). Data was tested for normal distribution using the Shapiro-Wilk test. Based on the assessment of normality, a paired sample t-test or its non-parametric equivalent was conducted to determine any learning effect or systematic bias between sample mean scores for the test and re-test. Between-group differences (i.e., pre-APHV and post-APHV) in single-leg hop test measures and inter-limb asymmetry were expressed as effect sizes, determined by calculating Cohen’s d. Cohen’s d is a measure that defines whether a difference is of practical value. Cohen’s d is classified as follows: 0.2 ≤ d ≤ 0.49 (small), 0.50 ≤ d ≤ 0.79 (moderate), and d ≥ 0.8 (large) [21]. Relative reliability was determined by calculating the intra-coeficient of correlation (ICC_(3,1))_. ICC is defined as intraclass correlation coefficient. An ICC_(3,1)_<0.50 was considered poor, 0.50 ≤ ICC_(3,1)_<0.75 moderate, 0.75 ≤ ICC_(3,1)_<0.90 good, and ICC_(3,1)_>0.90 excellent [22]. Absolute reliability was analyzed through the standard error of measurements (SEM) expressed as CV. It was calculated by dividing the SD of the difference between scores by the square root of 2 [23]. The Smallest Worthwhile Change (SWC) is commonly calculated as 0.2 × the between-subject standard deviation (SD) of the performance measure at baseline. This threshold corresponds to a small effect size based on Cohen’s convention and represents the minimum change considered practically meaningful. By comparing the smallest worthwhile change (SWC) with the SEM score, test sensitivity in detecting systematic variation in performance can be determined [24]. In the case SEM < SWC, the test’s capacity to detect small performance changes is considered “good”; when SEM = SWC, it is considered “OK”, and when SEM > SWC, it is rated as “marginal” [25]. The coefficient of variability is measured on a group level (SEM – SWC method) and not an individual level averaged on the group level. The level of significance for all tests was set at p < 0.05. All statistical analyses were conducted using SPSS v.24.0 for Windows (SPSS, Inc., Chicago, IL, USA).

## Results

Significant (all p < 0.001) between-group differences were found for chronological age and all examined anthropometric measures, except for the body fat (p > 0.05) (Table 1). Data regarding test-retest reliability with an inter-test interval of seven days is presented in Table 2.

**Table 2:** Performance variables for single leg hop test in the test and re-test measurements according to age at peak height velocity (APHV).

Characteristics	Pre-APHV (n = 34)	Post-APHV (n = 30)	Cohen’s d	*P* VALUE
**SLH-DL Test**	77.43 ± 28.66	100.83 ± 19.39	0.96*	0.0001
**SLH-DL Re-Test**	78.43 ± 27.96	99.90 ± 16.18	0.94*	0.0001
**SLH-NDL Test**	70.29 ± 26.95	101.50 ± 11.42	1.51*	0.0001
**SLH-NDL Re-Test**	72.09 ± 23.77	101.50 ± 9.93	1.61*	0.0001
**Inter-limb asymmetry Test**	5.44 ± 8.33	−1.05 ± 8.36	0.78*	0.01
**Inter-limb asymmetry Re-Test**	3.87 ± 12.43	−1.26 ± 7.82	0.49	0.04

Values are means and standard deviations. APHV = age at peak height velocity, *significantly different (p < 0.05) between pre-APHV and post-APHV, SLH-DL = single-leg hop test with dominant leg, SLH-NDL = single-leg hop test with non-dominant leg.

### Between-group differences in measures of single leg hop test with the dominant and non-dominant leg and interlimb asymmetry

Among the 64 eligible participants involved in this study, 34 were classified as pre-PHV (pre-adolescent peak height velocity), while 30 fell under the post-PHV (post-adolescent peak height velocity) category. Results presented in Table 2 demonstrated statistically significant differences, indicating that post-PHV individuals exhibited superior performance across almost all examined variables compared to pre-PHV participants (p < 0.05; range:0.000–0.04).

### Reliability and Sensitivity Outcomes

The data outcomes for the inter-limb asymmetry scores, pertaining to both the dominant and non-dominant leg, are presented in Table 2. The statistical analysis revealed good relative reliabilities for the inter-limb asymmetry scores, with ICC_(3,1)_ of 0.78 and 0.93 observed in prepubertal and post-pubertal male soccer players, respectively (Table 3). Moreover, regarding absolute reliabilities, the typical error of measurement recorded was less than 5% in both cohorts.

**Table 3:** The intra-class correlation coefficient, SEM, and SWC for inter-limb asymmetry score, single leg hop test for the dominant and non-dominant leg in prepubertal and post-pubertal male soccer players.

Group	Variables	ICC_(3,1)_	SEM	SEM%	SWC_0.2_
**Prepubertal (n = 34)**	**Dominant leg (cm)**	0.997	1.62	1.26	5.62
**Post-pubertal (n = 30)**	**Dominant leg (cm)**	0.966	2.77	2.81	3.54
**Prepubertal (n = 34)**	**Non-dominant leg (cm)**	0.970	5.49	3.91	5.05
**Postpubertal (n = 30)**	**Non-dominant leg (cm)**	0.915	1.91	0.20	2.12
**Prepubertal (n = 34)**	**Inter-limb asymmetry (%)**	0.778	5.94	0.28	2.11
**Postpubertal (n = 30)**	**Inter-limb asymmetry (%)**	0.925	1.82	0.02	2.12

ICC_(3,1)_: Intraclass correlation coefficient; SEM: Standard error of measurement; SWC_0.2_: smallest worthwhile change (0.2 standard deviations).

In terms of the dominant leg and non-dominant leg, the findings revealed good relative (all ICC_(3,1)_ >0.91) and absolute reliabilities (all SEM < 5%) in the prepubertal and post-pubertal group.

Regarding the sensitivity analysis, SEM was higher than the SWC (0.2), indicating a “marginal” ability of the inter-limb asymmetry scores test to detect small performance changes in the prepubertal group. However, the SEM was lower than the SWC (0.2), indicating the inter-limb asymmetry score was “good” for detecting small performances in post-pubertal male soccer players.

Regarding the dominant leg, the sensitivity analysis in the prepubertal group revealed that the SWC (0.2) was higher than the SEM in the prepubertal and post-puberal groups, indicating a good ability to detect small performance changes.

According to the non-dominant leg, calculations revealed that the SEM was higher than the SWC (0.2) in the prepubertal group, indicating the marginal ability of this test to detect small performance changes.

## Discussion

This study is the first to investigate the reliability and sensitivity of inter-limb asymmetry, assessed using the single-leg hop test, in male youth soccer players at different maturity stages. The primary findings demonstrate that both absolute and relative reliability were confirmed in pre- and post-APHV groups. Specifically, relative reliability was good in prepubertal players (ICC_(3,1)_=0.77) and excellent in post-pubertal players (ICC_(3,1)_= 0.92). These findings align with earlier work reporting strong reliability in unilateral strength assessments. For instance, Bishop et al. [26] observed low variability (CV = 5.44–5.70%) and excellent relative reliability (ICC = 0.93–0.94) for unilateral isometric squats in recreational adult athletes, while Spiteri et al. [27] reported acceptable absolute (CV = 5.5–7.0%) and near-perfect relative (ICC = 0.97) reliability for peak force. Collectively, these data support the notion that inter-limb asymmetry can be reliably assessed in soccer players.

Interestingly, greater between-limb differences were observed in the prepubertal group, which may reflect increased coordinative variability and lower motor-control stability commonly seen during early stages of development, rather than solely representing true neuromuscular asymmetry. This interpretation is consistent with recent evidence highlighting the interaction between dominant-limb preference, sport-specific movement patterns, and developmental coordination in young soccer players [28]. Nevertheless, evaluating asymmetry through a single test may not capture its full complexity, as asymmetry outcomes can vary according to task characteristics and loading conditions [29]. Therefore, asymmetry data should be interpreted cautiously, particularly in younger athletes, and within the specific context of the movement task being assessed.

Both the dominant and non-dominant leg demonstrated reliable performance in the single-leg hop test, consistent with previous evidence [19]. Although Bishop et al. [19] reported slightly higher absolute reliability (<5.8%) in single-leg countermovement jumps, both studies found similarly strong relative reliability (>0.85 ICC). The physical demands of the single-leg hop test may partly explain these findings, as the task requires coordinated force production, dynamic balance, and rapid neuromuscular control during both propulsion and landing phases [30]. Previous research has highlighted the importance of neuromuscular function, muscle stiffness regulation, and reactive force capabilities during explosive lower-limb actions in youth athletes [31]. In particular, muscles such as the gastrocnemius, rectus femoris, and biceps femoris contribute substantially to force generation and landing stabilization during unilateral hopping tasks [32].

Another notable finding is that maturation did not appear to significantly affect the reliability of inter-limb asymmetry. While adolescents often experience temporary decrements in coordination and motor control during rapid growth, especially around PHV [7], this study found reliable asymmetry measures in both maturity groups. These results suggest that, although growth may temporarily disrupt motor skills, inter-limb asymmetry remains a stable and measurable characteristic across pre- and post-APHV stages. Nonetheless, the absence of a circa-PHV group in this study limits conclusions about players undergoing their peak growth phase, when asymmetry may be most volatile.

These maturity-dependent responses may also reflect “synergistic adaptations,” whereby biological maturation interacts with training-induced changes [33]. Supporting this view, prior work has shown that sprinting asymmetries in school-aged boys were largely unaffected by maturation [34]. Together, these findings indicate that inter-limb asymmetry, at least in hopping tasks, stabilizes after early adolescence and can be reliably monitored throughout youth development.

Several limitations should be acknowledged. First, the study design excluded circa-PHV athletes, who may present distinct asymmetry patterns. Second, only one functional test (single-leg hop) was used, limiting the generalizability of findings across other performance domains (e.g., sprinting or change of direction). Third, only male players were assessed, precluding inferences for female athletes. Fourth, the cross-sectional design does not permit conclusions regarding longitudinal changes or causality. Additionally, no neurophysiological tools (e.g., electromyography) were included to clarify the underlying mechanisms of asymmetry. Fifth, given the sensitivity of asymmetry ratios and the relatively high variability, reliance on just two trials may not fully capture maximal performance, potentially underestimating asymmetry magnitudes. A final limitation of the present study is that factors such as verbal instructions, movement goals, and attentional focus during the single-leg hop test were not systematically controlled beyond standardized task instructions. Previous evidence has shown that these factors may influence lower-limb ballistic performance and performance variability, potentially affecting SEM and SWC values [35]. Future studies should further standardize and report these methodological aspects to improve the consistency and interpretability of asymmetry assessments.

## Conclusion

This study demonstrates that inter-limb asymmetry assessed via the single-leg hop test shows acceptable reliability and sensitivity in both pre- and post-pubertal male soccer players. Reliability was evident for asymmetry measures across maturity groups, with values meeting commonly accepted thresholds for relative and absolute reliability. Furthermore, maturation status did not appear to substantially influence the reliability of the asymmetry scores, suggesting that the single-leg hop test may provide a consistent method for assessing inter-limb asymmetry across different developmental stages.

Sensitivity analysis indicated that the ability to detect small changes differed between maturity groups, with better sensitivity observed in post-pubertal players compared with prepubertal players. These findings contribute to the methodological understanding of SLHT-derived inter-limb asymmetry assessment in youth soccer populations.

Future research should examine players around peak height velocity (circa-PHV), incorporate additional asymmetry assessment modalities, and explore longitudinal changes in asymmetry measures across maturation to further clarify the utility and responsiveness of these assessments in youth athletes.

## Supporting information

S1 Data Raw Data-Asymetry-Plos One. (XLSX)
