# Supplementary material for: Test–retest reliability and sensitivity of horizontal jump inter-limb asymmetry in youth soccer players across maturity stages
Source: PLoS One. 2026 Jun 22;21(6):e0341344. doi: 10.1371/journal.pone.0341344 (PMC13286214; doi:10.1371/journal.pone.0341344)
Supplement: Attachment — Submitted filename: Point-by-point revisions-R1.pdf [file pone.0341344.s002.pdf]

Plos One
Manuscript: PONE-D-25-55309
Title: Inter-limb asymmetry during horizontal jump test in youth soccer: Evaluating reliable and sensitive metrics through maturation
First revision

Dear Editor,
Dear Reviewers,
We would like to express our gratitude for your valuable time you have invested in our manuscript and the constructive and helpful comments on the revised paper. We have responded to all of your queries in point-by-point responses. Whenever needed, we have made changes to the re-revised manuscript. We hope that you will find the current version of our manuscript suitable for publication in Plos One.

Kind regards

Prof. Raouf Hammami, PhD
On behalf of all co-authors

Reviewer #1: Changes are highlighted in yellow

The article is well written and logically structured, addressing a highly relevant topic in sport science. The rationale and introduction are clearly articulated, providing a strong foundation for the study. The statistical analyses are well executed and appropriately interpreted, supporting the validity of the findings. Overall, this is a well-conducted and insightful piece of research. However, i have some points that should be take into consideration.

Authors' response: We thank the reviewer for their positive evaluation of our work and for acknowledging the clarity, structure, and relevance of the manuscript. We appreciate the recognition of the methodological rigor and the appropriateness of our statistical analyses. We also thank the reviewer for the constructive points raised. In the revised version, we have carefully considered each of the issues highlighted and addressed them accordingly to further strengthen the manuscript. Detailed responses to each comment are provided below.

Line 41: Please provide more details about the prepuberal and postpuberal participants (i.e., Body mass; heigh; age. Maturity offset)

Authors' response: Included as follow:

"Participants were divided into pre-pubertal (n = 34; age: 8.1 ± 1.4 years; body mass: 35.7 ± 3.4 kg; height: 122 ± 9 cm; maturity offset: −2.38 ± 0.83 years) and a post-

pubertal (n = 30; age: 16.5 ± 0.3 years; body mass: 39.6 ± 6.1 kg; height: 154 ± 7 cm; maturity offset: 1.17 ± 0.45 years) groups based on individual peak height velocity."

Line 92: pre-pubertal should be prepuberal please make changes through the whole manuscript

Authors' response: Revised through the manuscript. Thank you.

Statistical analysis please provide the Type of ICC: I think it ill be ICC (3,1) Please provide how to calculate the SWC

Authors' response: Included as follow:

"The Smallest Worthwhile Change (SWC) is commonly calculated as 0.2 × the between-subject standard deviation (SD) of the performance measure at baseline. This threshold corresponds to a small effect size based on Cohen's convention and represents the minimum change considered practically meaningful."

Line 222: Nice i found that you put the type of ICC (3,1). So add it inthe statistical analysis
Authors' response: Revised through the statistical procedure section. Thank you.

Discussion
Line 242-244: to investigate better than to examine (use neutral objective language) assessed using is more formal that assessed via

Authors' response: Revised as follow:

"This study is the first to investigate the reliability and sensitivity of inter-limb asymmetry, assessed using the single-leg hop test, in male youth soccer players at different maturity stages."

Line 246: ICC = 0.778: I recommend standardizing all numerical values to two decimal places throughout the manuscript

Authors' response: We thank the reviewer for this helpful suggestion. In accordance with the recommendation, we have standardized all numerical values throughout the manuscript to two decimal places, ensuring consistency and improving readability.

Reviewer #2: Changes are highlighted in blue

Abstract
Reduce repetitiveness. For example:

Lines 41-46 "Participants were divided into pre-pubertal and post-pubertal groups based on individual peak height velocity. Participants completed the single-leg hop test to predict and identify interlimb asymmetries. This test involved participants hopping on each leg separately, and the maximal distance achieved was recorded. The reliability of the inter-limb asymmetry was calculated for pre-pubertal and post-pubertal groups. The reliability of the inter-limb asymmetry was calculated according to peak height velocity"
Perhaps change to:

"The reliability of the inter-limb asymmetry was calculated for pre-pubertal and post-pubertal groups based on individual peak height velocity. This test involved participants hopping on each leg separately, and the maximal distance achieved was recorded."
Authors' response: Revised and changed as suggested. Thank you.

Line 56-57: Authors state: "Thus, this may imply transfer effects to strength/power training and vice versa." You start this again a few lines later. I would delete this sentence to reduce repetitiveness.

Authors' response: This sentence was deleted as suggested. Thank you.

Key words are words used to expand search criteria for readers. If the word is in the title, it does not need to be repeated in key words. For example: youth, soccer, etc.

Authors' response: The key words were now changed to the following:

"Keywords: young; football; imbalance; power training, peak height velocity"

Methods
Lines 126-128: You must have inputted more parameters to calculate the sample size. For example what was the test family, statistical test, project effect size, correlation among measures, number of groups and measurements, etc.? Or was the estimate based on means and SD from prior similar studies? If so provide the references.

Authors' response: We thank the reviewer for this insightful comment. We now provide detailed information on the parameters used for the a priori sample size calculation. The sample size was estimated using G*Power (version 3.1.9.7) based on a mixed-model ANOVA design (test family: F-tests; statistical test: repeated-measures, within–between interaction). The calculation assumed two groups (prepubertal vs. post-pubertal) and two measurement time points. A medium projected effect size ($f = 0.25$), an α level of 0.05, a desired power of 0.80, and a correlation among repeated measures of 0.50 were entered. These values were selected based on previously reported effect sizes and variability in youth soccer and maturity-related training studies (Hammami et

al., 2020; Moran et al., 2017; Radnor et al., 2018). The analysis indicated a minimum required sample of 52 participants; therefore, 64 players were recruited to accommodate potential dropouts. All details have now been added to the Methods section as follow:

"The sample size was determined a priori using G*Power version 3.1.9.7 (University of Düsseldorf, Germany). The calculation was based on a mixed-model ANOVA (test family: F-tests; statistical test: repeated-measures, within–between interaction), with two groups (prepubertal vs. post-pubertal) and two measurement time points. A projected medium effect size (f = 0.25), an α level of 0.05, a desired statistical power of 0.80, and an assumed correlation among repeated measures of 0.50 were used. These parameters were derived from reported effect sizes and variability in previous youth soccer and maturity-related training interventions (Hammami et al., 2020; Moran et al., 2017; Radnor et al., 2018). The analysis indicated that a minimum of 52 participants was required; therefore, 64 players were recruited to account for potential attrition."

References used:

- Hammami, R., Chaouachi, A., Makhlouf, I., Granacher, U., & Behm, D. G. (2020). Associations between change of direction, speed, jump, and anthropometric measurements in youth soccer players. Journal of Strength and Conditioning Research, 34(3), 802–812.

- Moran, J., Sandercock, G., & Rumpf, M. (2017). The effects of maturation on measures of asymmetry, speed, and power in youth soccer players. Journal of Sports Sciences, 35(18), 1798–1804.

- Radnor, J. M., Oliver, J. L., Waugh, C. M., Myer, G. D., Moore, I. S., & Lloyd, R. S. (2018). The influence of maturity status on movement quality in adolescent male athletes. Journal of Strength and Conditioning Research, 32(2), 548–556.

Line 181: Was the acronym ICC defined previously. If not, then it needs to be defined the first time it appears.

References used: Defined as suggested. Thank you.

Line 185: Have you previously defined how SWC is calculated. If I remember correctly it is the performance SD x the effect size of interest (e.g., small effect size would = 0.2). The reader needs to know how SWC is calculated.

References used: Defined as suggested. Thank you.

Results

Line 196-197: Authors state: "except for inter-limb asymmetry during the re-test, which was borderline significant (Table 1)." What is borderline significant (p=0.06 or p=0.1

or p=0.2???). You refer to Table 1 but there are no p values provided in table 1 so we have no idea what borderline means.

Authors' responses: Dear Reviewer, we appreciate your insightful remark. In response, we have revised the sentence and added the p-value to Table 1 as suggested.

Table 2 : Please provide all the precise p values not just that all were $p<0.05$. Lines 208-210: Provide the range of p values.

Authors' responses: All details were added as suggested. Thank you

Line 215: Commendable is not a standard description of the strength of the reliability scores. Typically, as you state in the statistical analysis section they are termed as excellent, good, moderate etc.

Authors' responses: We thank the reviewer for this observation. We agree that "commendable" is not a standard descriptor for reliability outcomes. In accordance with the terminology used in the statistical analysis section, we have replaced "commendable" with standardized reliability classifications (i.e., *excellent, good, moderate,* or *poor*) based on the corresponding ICC thresholds. The text has been updated accordingly to ensure consistency and adherence to accepted reporting standards.

Discussion
Well written and organized.

Authors' responses: We thank the reviewer for the positive feedback and appreciation of the manuscript's organization and clarity.
