# Supplementary material for: Test–retest reliability and sensitivity of horizontal jump inter-limb asymmetry in youth soccer players across maturity stages
Source: PLoS One. 2026 Jun 22;21(6):e0341344. doi: 10.1371/journal.pone.0341344 (PMC13286214; doi:10.1371/journal.pone.0341344)
Supplement: Attachment — Submitted filename: Point-bypoint revisions-R2.pdf [file pone.0341344.s003.pdf]

**Plos One Manuscript: PONE-D-25-55309**

**Title: Inter-limb asymmetry during horizontal jump test in youth soccer: Evaluating reliable and sensitive metrics through maturation**

**Second revision**

**Dear Editor,**

**Dear Reviewers,**

We would like to express our gratitude for your valuable time you have invested in our manuscript and the constructive and helpful comments on the revised paper. We have responded to all of your queries in point-by-point responses. Whenever needed, we have made changes to the re-revised manuscript. We hope that you will find the current version of our manuscript suitable for publication in Plos One.

Kind regards

**Prof. Raouf Hammami,** PhD On behalf of all co-authors

**Reviewer #1**

Thank you for thoroughly addressing and responding to all the comments. The article is now ready for publication.

**Authors' response:** We sincerely thank the Editor and Reviewers for their time and valuable feedback. We have carefully addressed all comments and are grateful for the positive assessment that the manuscript is now ready for publication.

**Reviewer #2:** Changes are highlighted in yellow

Authors have addressed most of my prior suggestion's. There are a couple of very minor revisions needed:
1. The proper term is prepubertal not prepuberal (add the letter "t'). This misspelling occurs throughout the manuscript.

**Authors' response:** Thank you for pointing this out. The term has been corrected from prepuberal to prepubertal throughout the entire manuscript.

2. ICC is defined as intraclass correlation coefficient: p. 8 line 189

**Authors' response:** We thank the Reviewer for this comment. The definition of ICC has been included at p. 8, line 189.

3. Line 226: Change unveiled to revealed.

**Authors' response:** Thank you for this suggestion. The term unveiled has been corrected to revealed in the manuscript.
