# Supplementary material for: Test–retest reliability and sensitivity of horizontal jump inter-limb asymmetry in youth soccer players across maturity stages
Source: PLoS One. 2026 Jun 22;21(6):e0341344. doi: 10.1371/journal.pone.0341344 (PMC13286214; doi:10.1371/journal.pone.0341344)
Supplement: Attachment — Submitted filename: Response R3-Plos One.pdf [file pone.0341344.s004.pdf]

Plos One

Manuscript: PONE-D-25-55309

Title: Test–Retest Reliability and Sensitivity of Horizontal Jump Inter-Limb Asymmetry in Youth Soccer Players Across Maturity Stages

Third revision

Dear Editor,

Dear Reviewers,

We would like to express our gratitude once again for your valuable time you have invested in our manuscript and the constructive and helpful comments on the revised paper. We have responded to all of your queries in point-by-point responses. Whenever needed, we have made changes to the re-revised manuscript. We hope that you will find the current version of our manuscript suitable for publication in Plos One.

Kind regards

Prof. Raouf Hammami, PhD On behalf of all co-authors

Reviewer #1: I would like to congratulate the authors for their careful revision of the manuscript. They have addressed my comments and recommendations appropriately, and the responses provided are clear and satisfactory. The changes made have improved the quality and clarity of the paper. Thank you for your efforts in revising the manuscript.

Authors' response: We sincerely thank the reviewer for the positive evaluation of our revised manuscript. We greatly appreciate your careful assessment and encouraging comments. We are pleased that our revisions and responses have satisfactorily addressed your concerns and that the changes made have improved the quality and clarity of the paper. Your valuable feedback has been highly helpful in strengthening the manuscript. Thank you again for your time and consideration.

Reviewer #2: Authors have addressed my second round of requested revisions.

Authors' response: We sincerely thank the reviewer for the careful evaluation of our revised manuscript. We appreciate your recognition that the requested second-round revisions have been adequately addressed. Your constructive feedback has been invaluable in improving the quality of our work. Thank you again for your time and consideration.

Reviewer #3: Changes are highlighted in yellow

I would like to thank the authors for the opportunity to review this manuscript. The study addresses a relevant and timely question in youth soccer research, applying a sound test–retest design to examine the reliability and sensitivity of inter-limb asymmetry across maturity stages. The clear stratification by APHV, the use of established reliability and sensitivity metrics (ICC, SEM, SWC),

and the practical orientation of the work are notable strengths. The points below are intended as constructive suggestions to further strengthen the methodological transparency and the interpretive precision of the manuscript.

Authors' response: We sincerely thank the reviewer for the thorough and thoughtful evaluation of our manuscript. We greatly appreciate your positive comments regarding the relevance and timeliness of the study, as well as your recognition of the methodological strengths of our work, including the test–retest design, the stratification by APHV, and the use of established reliability and sensitivity metrics (ICC, SEM, and SWC). We are also grateful for your acknowledgment of the practical relevance of the study for youth soccer research and practice.

We appreciate the constructive suggestions provided, which have helped us improve the methodological transparency and interpretive precision of the manuscript. All comments have been carefully considered and addressed in the revised version, and we believe that these revisions have further strengthened the quality and clarity of the paper. Thank you again for your valuable feedback and time devoted to reviewing our work.

General comment

The title and several passages in the Abstract and Discussion frame inter-limb asymmetry as an indicator for "prescribing power training priorities" and for "designing targeted training interventions." However, the cross-sectional design — without injury or longitudinal performance outcomes — does not support inferences about training prescription, transfer effects, or injury-related implications. In line with recent commentary on this issue (Afonso et al., 2026), I recommend aligning the title and conclusions with what the data actually address: test–retest reliability and sensitivity of the SLHT-derived asymmetry score across two maturity groups.

Authors' response: We thank the reviewer for this important and insightful comment. In accordance with your recommendation and the recent commentary by Afonso et al. (2026), we revised the title, Abstract, Discussion, and Conclusion to better align with the scope of the study and the cross-sectional nature of the design. Specifically, we removed statements implying training prescription, transfer effects, or injury-related implications and focused the interpretation on the test–retest reliability and sensitivity of the SLHT-derived inter-limb asymmetry scores across maturity groups.

Participant classification

The competitive level of the players is not specified. I recommend classifying the sample according to McKay et al. (2022), ideally applying the scoring criteria operationalised by Wilkins et al. (2025) to support the reliability of that classification and the generalisability of the findings.

Authors' response: We thank the reviewer for this valuable suggestion. In the revised manuscript, we have now clarified the competitive level of the participants and classified the sample in accordance with the framework proposed by McKay et al. (2022). In addition, we applied the

operationalised scoring criteria outlined by Wilkins et al. (2025) to ensure a more transparent and standardized classification of player level. These additions have been included in the Methods section to improve clarity, reproducibility, and the generalisability of the findings.

Order of limb testing

It is not stated whether the order of testing between the dominant and non-dominant limb was randomised, counterbalanced, or fixed. Given that the asymmetry score is computed directly from the between-limb difference, this detail should be clarified.

Authors' response: We thank the reviewer for this important point. The order of testing between limbs was kept fixed and identical for all participants and both testing sessions to ensure standardisation of measurement procedures. This clarification has been added to the Methods section.

Movement goal and attentional focus

The authors report that participants were instructed to "execute a maximal forward hop," without further detail on verbal instructions, movement goal, or attentional focus. Evidence indicates that these factors meaningfully affect lower-limb ballistic performance and its variability (Pompa et al., 2026), with direct implications for the SEM and SWC values reported here. Please specify the exact instructions used, or acknowledge this among the limitations.

Authors' response: We thank the reviewer for this valuable observation. In the study limitation, we have clarified the standardized verbal instructions provided during the single-leg hop test, including the requirement to perform a maximal forward hop while maintaining balance upon landing. We also acknowledge that factors such as verbal cueing, movement goal, and attentional focus may influence ballistic performance variability and, consequently, the SEM and SWC outcomes, as highlighted by Pompa et al. (2026). This consideration has now been included in the limitations section.

Swing leg and propulsion strategy

It is not clarified whether participants were allowed a swing-leg drive or whether this was constrained. An unrestricted swing-leg action increases forward momentum and may differentially affect dominant vs. non-dominant performance, particularly in younger players. Please specify the procedure.

Authors' response: We thank the reviewer for this important comment. We have now clarified the propulsion strategy used during the single-leg hop test. Specifically, participants kept their hands on their hips throughout the movement, while a natural swing-leg action was permitted to allow coordinated execution of the hop. This information has been added to the Methods section.

Asymmetry vs. coordinative differences in younger players

The Discussion states that "asymmetry appeared more pronounced in younger players, consistent with previous findings that asymmetries decline with maturation." The greater between-limb difference observed in the prepubertal group may, however, reflect inter-limb coordinative variability and lower motor-control stability rather than a true neuromuscular asymmetry. This distinction carries different practical implications, and recent work has discussed how dominant-limb preference, repetitive sport-specific gestures, and developmental coordination interact in young soccer players (D'Onofrio et al., 2025). I recommend reframing this passage accordingly.

Authors' response: We thank the reviewer for this insightful comment. In response, we revised the Discussion to avoid interpreting greater between-limb differences in younger players solely as neuromuscular asymmetry. Instead, we now acknowledge that these findings may also reflect increased coordinative variability and reduced motor-control stability during early development, consistent with the perspective proposed by D'Onofrio et al. (2025).

Stretch-shortening cycle and citation support

The statement that "the hop test heavily recruits the stretch-shortening cycle, which enhances elastic energy storage and release, allowing for more forceful concentric actions [35–38]" is not substantively supported by the cited references. Hammami et al. (2023) discuss SSC efficiency generically as a training adaptation, without characterising the SLHT as an SSC-dependent task; Andrade et al. (2020) examined bilateral drop jumps in volleyball players, with only speculative reference to elastic energy; Radnor et al. (2020) addresses muscle architecture across maturity, not SSC mechanics in hop tests; and Taube et al. (2012) investigated neuromuscular adaptations to drop jump training, focusing on reflex contributions and joint stiffness. I recommend either reformulating the statement to match what the cited sources report, or replacing the citations with primary sources specifically characterising the biomechanics of single-leg hop tests.

Authors' response: We thank the reviewer for this suggestion. We have revised the statement to better align with the literature and have supported it with appropriate references, which describe the neuromuscular demands of unilateral hop-based tasks in terms of coordinated force production, dynamic balance, and neuromuscular control during propulsion and landing phases.

Suggested references

Afonso J, Andrade R, Oliveira E, Turner A, Warneke K, Bishop C. Asymmetries and injury risk: when titles spin the tale. J Strength Cond Res. 2026;40(5):e530–e533. doi:10.1519/JSC.0000000000005430
D'Onofrio R, Perna P, Pompa D, Civitillo C, Sannicandro I, Manzi V. Asymmetry, lumbo-pelvic hip complex and injury in European soccer players. J Sports Med Phys Fitness. 2025;65(0):000–000. doi:10.23736/S0022-4707.25.16845-X
McKay AKA, Stellingwerff T, Smith ES, et al. Defining training and performance caliber: a participant classification framework. Int J Sports Physiol Perform. 2022;17(2):317–331.
Wilkins L, et al. Reliability of participant classification in sport and exercise science: application of McKay et al.'s (2022) framework. J Sports Sci. 2025;43:2914–2926.

Pompa D, Carson HJ, Romagnoli R, et al. Effect of movement goal on countermovement jump performance in athletes across different sports. Sci Rep. 2026;16:9748. doi:10.1038/s41598-026-40260-9
