# Supplementary material for: Test–retest reliability and sensitivity of horizontal jump inter-limb asymmetry in youth soccer players across maturity stages
Source: PLoS One. 2026 Jun 22;21(6):e0341344. doi: 10.1371/journal.pone.0341344 (PMC13286214; doi:10.1371/journal.pone.0341344)
Supplement: Peer review history

# Peer review: Decision Letter 0

**DOI:** 10.1371/journal.pone.0341344.r001

**Authors:** Holakoo Mohsenifar

12 Nov 2025

PONE-D-25-55309

Inter-limb asymmetry during horizontal jump test in youth soccer: Evaluating reliable and sensitive metrics through maturation

PLOS ONE

Dear Dr. Raouf Hammami,

Thank you for submitting your manuscript to PLOS ONE. After careful consideration, we feel that it has merit but does not fully meet PLOS ONE’s publication criteria as it currently stands. Therefore, we invite you to submit a revised version of the manuscript that addresses the points raised during the review process.

Please submit your revised manuscript by Dec 27 2025 11:59PM. If you will need more time than this to complete your revisions, please reply to this message or contact the journal office at plosone@plos.org. When you're ready to submit your revision, log on to https://www.editorialmanager.com/pone/ and select the 'Submissions Needing Revision' folder to locate your manuscript file.

Please include the following items when submitting your revised manuscript:

- A rebuttal letter that responds to each point raised by the academic editor and reviewer(s). You should upload this letter as a separate file labeled 'Response to Reviewers'.
- A marked-up copy of your manuscript that highlights changes made to the original version. You should upload this as a separate file labeled 'Revised Manuscript with Track Changes'.
- An unmarked version of your revised paper without tracked changes. You should upload this as a separate file labeled 'Manuscript'.

-->If you would like to make changes to your financial disclosure, please include your updated statement in your cover letter. Guidelines for resubmitting your figure files are available below the reviewer comments at the end of this letter.

If applicable, we recommend that you deposit your laboratory protocols in protocols.io to enhance the reproducibility of your results. Protocols.io assigns your protocol its own identifier (DOI) so that it can be cited independently in the future. For instructions see: https://journals.plos.org/plosone/s/submission-guidelines#loc-laboratory-protocols. Additionally, PLOS ONE offers an option for publishing peer-reviewed Lab Protocol articles, which describe protocols hosted on protocols.io. Read more information on sharing protocols at https://plos.org/protocols?utm_medium=editorial-email&utm_source=authorletters&utm_campaign=protocols.

We look forward to receiving your revised manuscript.

Kind regards,

Holakoo Mohsenifar

Academic Editor

PLOS ONE

Journal Requirements:

When submitting your revision, we need you to address these additional requirements.

1. Please ensure that your manuscript meets PLOS ONE's style requirements, including those for file naming. The PLOS ONE style templates can be found at

https://journals.plos.org/plosone/s/file?id=wjVg/PLOSOne_formatting_sample_main_body.pdf and

https://journals.plos.org/plosone/s/file?id=ba62/PLOSOne_formatting_sample_title_authors_affiliations.pdf.

2. In the online submission form you indicate that your data is not available for proprietary reasons and have provided a contact point for accessing this data. Please note that your current contact point is a co-author on this manuscript. According to our Data Policy, the contact point must not be an author on the manuscript and must be an institutional contact, ideally not an individual. Please revise your data statement to a non-author institutional point of contact, such as a data access or ethics committee, and send this to us via return email. Please also include contact information for the third party organization, and please include the full citation of where the data can be found.

3, Your ethics statement should only appear in the Methods section of your manuscript. If your ethics statement is written in any section besides the Methods, please delete it from any other section.

4. If the reviewer comments include a recommendation to cite specific previously published works, please review and evaluate these publications to determine whether they are relevant and should be cited. There is no requirement to cite these works unless the editor has indicated otherwise.

5. Please review your reference list to ensure that it is complete and correct. If you have cited papers that have been retracted, please include the rationale for doing so in the manuscript text, or remove these references and replace them with relevant current references. Any changes to the reference list should be mentioned in the rebuttal letter that accompanies your revised manuscript. If you need to cite a retracted article, indicate the article’s retracted status in the References list and also include a citation and full reference for the retraction notice.

[Note: HTML markup is below. Please do not edit.]

Reviewers' comments:

Reviewer's Responses to Questions

**Comments to the Author**

1. Is the manuscript technically sound, and do the data support the conclusions?

The manuscript must describe a technically sound piece of scientific research with data that supports the conclusions. Experiments must have been conducted rigorously, with appropriate controls, replication, and sample sizes. The conclusions must be drawn appropriately based on the data presented.

Reviewer #1: Yes

Reviewer #2: Yes

**********

2. Has the statistical analysis been performed appropriately and rigorously?

Reviewer #1: Yes

Reviewer #2: Yes

**********

3. Have the authors made all data underlying the findings in their manuscript fully available?

The PLOS Data policy requires authors to make all data underlying the findings described in their manuscript fully available without restriction, with rare exception (please refer to the Data Availability Statement in the manuscript PDF file). The data should be provided as part of the manuscript or its supporting information, or deposited to a public repository. For example, in addition to summary statistics, the data points behind means, medians and variance measures should be available. If there are restrictions on publicly sharing data—e.g. participant privacy or use of data from a third party—those must be specified.

Reviewer #1: Yes

Reviewer #2: Yes

**********

4. Is the manuscript presented in an intelligible fashion and written in standard English?

PLOS ONE does not copyedit accepted manuscripts, so the language in submitted articles must be clear, correct, and unambiguous. Any typographical or grammatical errors should be corrected at revision, so please note any specific errors here.

Reviewer #1: Yes

Reviewer #2: Yes

**********

5. Review Comments to the Author

Please use the space provided to explain your answers to the questions above. You may also include additional comments for the author, including concerns about dual publication, research ethics, or publication ethics. (Please upload your review as an attachment if it exceeds 20,000 characters)

Reviewer #1: The article is well written and logically structured, addressing a highly relevant topic in sport science. The rationale and introduction are clearly articulated, providing a strong foundation for the study. The statistical analyses are well executed and appropriately interpreted, supporting the validity of the findings. Overall, this is a well-conducted and insightful piece of research. However, i have some points that should be take into consideration.

Line 41: Please provide more details about the prepuberal and postpuberal participants (i.e., Body mass; heigh; age. Maturity offset)

Line 92: pre-pubertal should be prepuberal please make changes through he whole manuscript

Statistical analysis please provide te Type of ICC: I think it ill be ICC (3,1)

Please provide how to calculate the SWC

Line 222: Nice i found that you put the tyoe of ICC (3,1). So add it inthe statistical analysis

Discussion

Line 242-244: to investigate better than to examine (use neutral objective language)

assessed using is more formal tha assessed via

Line 246: ICC = 0.778: I recommend standardizing all numerical values to two decimal places throughout the manuscript

Reviewer #2: Abstract

Reduce repetitiveness. For example:

Lines 41-46 “Participants were divided into pre-pubertal and post-pubertal groups based on individual peak height velocity. Participants completed the single-leg hop test to predict and identify interlimb asymmetries. This test involved participants hopping on each leg separately, and the maximal distance achieved was recorded. The reliability of the inter-limb asymmetry was calculated for pre-pubertal and post-pubertal groups. The reliability of the inter-limb asymmetry was calculated according to peak height velocity”

Perhaps change to:

“The reliability of the inter-limb asymmetry was calculated for pre-pubertal and post-pubertal groups based on individual peak height velocity. This test involved participants hopping on each leg separately, and the maximal distance achieved was recorded.”

Line 56-57: Authors state: “Thus, this may imply transfer effects to strength/power training and vice versa.” You start this again a few lines later. I would delete this sentence to reduce repetitiveness.

Key words are words used to expand search criteria for readers. If the word is in the title, it does not need to be repeated in key words. For example: youth, soccer, etc.

Methods

Lines 126-128: You must have inputted more parameters to calculate the sample size. For example what was the test family, statistical test, project effect size, correlation among measures, number of groups and measurements, etc.? Or was the estimate based on means and SD from prior similar studies? If so provide the references.

Line 181: Was the acronym ICC defined previously. If not, then it needs to be defined the first time it appears.

Line 185: Have you previously defined how SWC is calculated. If I remember correctly it is the performance SD x the effect size of interest (e.g., small effect size would = 0.2). The reader needs to know how SWC is calculated.

Results

Line 196-197: Authors state: “except for inter-limb asymmetry during the re-test, which was borderline significant (Table 1).” What is borderline significant (p=0.06 or p=0.1 or p=0.2???). You refer to Table 1 but there are no p values provided in table 1 so we have no idea what borderline means.

Table 2 : Please provide all the precise p values not just that all were p<0.05.

Lines 208-210: Provide the range of p values.

Line 215: Commendable is not a standard description of the strength of the reliability scores. Typically, as you state in the statistical analysis section they are termed as excellent, good, moderate etc.

Discussion

Well written and organized.

**********

6. PLOS authors have the option to publish the peer review history of their article (what does this mean?). If published, this will include your full peer review and any attached files.

If you choose “no”, your identity will remain anonymous but your review may still be made public.

**Do you want your identity to be public for this peer review?**   For information about this choice, including consent withdrawal, please see our Privacy Policy.

Reviewer #1: No

Reviewer #2: **Yes:**   David Behm

**********

[NOTE: If reviewer comments were submitted as an attachment file, they will be attached to this email and accessible via the submission site. Please log into your account, locate the manuscript record, and check for the action link "View Attachments". If this link does not appear, there are no attachment files.]

To ensure your figures meet our technical requirements, please review our figure guidelines: https://journals.plos.org/plosone/s/figures

You may also use PLOS’s free figure tool, NAAS, to help you prepare publication quality figures: https://journals.plos.org/plosone/s/figures#loc-tools-for-figure-preparation.

NAAS will assess whether your figures meet our technical requirements by comparing each figure against our figure specifications.

# Author comment: Author response to Decision Letter 1

**DOI:** 10.1371/journal.pone.0341344.r002

10 Dec 2025

Plos One

Manuscript: PONE-D-25-55309

Title: Inter-limb asymmetry during horizontal jump test in youth soccer: Evaluating reliable and sensitive metrics through maturation

First revision

Dear Editor,

Dear Reviewers,

We would like to express our gratitude for your valuable time you have invested in our manuscript and the constructive and helpful comments on the revised paper. We have responded to all of your queries in point-by-point responses. Whenever needed, we have made changes to the re-revised manuscript. We hope that you will find the current version of our manuscript suitable for publication in Plos One.

Kind regards

Prof. Raouf Hammami, PhD

On behalf of all co-authors

Reviewer #1: Changes are highlighted in yellow

The article is well written and logically structured, addressing a highly relevant topic in sport science. The rationale and introduction are clearly articulated, providing a strong foundation for the study. The statistical analyses are well executed and appropriately interpreted, supporting the validity of the findings. Overall, this is a well-conducted and insightful piece of research. However, i have some points that should be take into consideration.

Authors’ response: We thank the reviewer for their positive evaluation of our work and for acknowledging the clarity, structure, and relevance of the manuscript. We appreciate the recognition of the methodological rigor and the appropriateness of our statistical analyses. We also thank the reviewer for the constructive points raised. In the revised version, we have carefully considered each of the issues highlighted and addressed them accordingly to further strengthen the manuscript. Detailed responses to each comment are provided below.

Line 41: Please provide more details about the prepuberal and postpuberal participants (i.e., Body mass; heigh; age. Maturity offset)

Authors’ response: Included as follow:

“Participants were divided into pre-pubertal (n = 34; age: 8.1 ± 1.4 years; body mass: 35.7 ± 3.4 kg; height: 122 ± 9 cm; maturity offset: −2.38 ± 0.83 years) and a post-pubertal (n = 30; age: 16.5 ± 0.3 years; body mass: 39.6 ± 6.1 kg; height: 154 ± 7 cm; maturity offset: 1.17 ± 0.45 years) groups based on individual peak height velocity.”

Line 92: pre-pubertal should be prepuberal please make changes through the whole manuscript

Authors’ response: Revised through the manuscript. Thank you.

Statistical analysis please provide the Type of ICC: I think it ill be ICC (3,1)

Please provide how to calculate the SWC

Authors’ response: Included as follow:

“The Smallest Worthwhile Change (SWC) is commonly calculated as 0.2 × the between-subject standard deviation (SD) of the performance measure at baseline. This threshold corresponds to a small effect size based on Cohen’s convention and represents the minimum change considered practically meaningful.”

Line 222: Nice i found that you put the type of ICC (3,1). So add it inthe statistical analysis

Authors’ response: Revised through the statistical procedure section. Thank you.

Discussion

Line 242-244: to investigate better than to examine (use neutral objective language)

assessed using is more formal that assessed via

Authors’ response: Revised as follow:

“This study is the first to investigate the reliability and sensitivity of inter-limb asymmetry, assessed using the single-leg hop test, in male youth soccer players at different maturity stages.”

Line 246: ICC = 0.778: I recommend standardizing all numerical values to two decimal places throughout the manuscript

Authors’ response: We thank the reviewer for this helpful suggestion. In accordance with the recommendation, we have standardized all numerical values throughout the manuscript to two decimal places, ensuring consistency and improving readability.

Reviewer #2: Changes are highlighted in blue

Abstract

Reduce repetitiveness. For example:

Lines 41-46 “Participants were divided into pre-pubertal and post-pubertal groups based on individual peak height velocity. Participants completed the single-leg hop test to predict and identify interlimb asymmetries. This test involved participants hopping on each leg separately, and the maximal distance achieved was recorded. The reliability of the inter-limb asymmetry was calculated for pre-pubertal and post-pubertal groups. The reliability of the inter-limb asymmetry was calculated according to peak height velocity”

Perhaps change to:

“The reliability of the inter-limb asymmetry was calculated for pre-pubertal and post-pubertal groups based on individual peak height velocity. This test involved participants hopping on each leg separately, and the maximal distance achieved was recorded.”

Authors’ response: Revised and changed as suggested. Thank you.

Line 56-57: Authors state: “Thus, this may imply transfer effects to strength/power training and vice versa.” You start this again a few lines later. I would delete this sentence to reduce repetitiveness.

Authors’ response: This sentence was deleted as suggested. Thank you.

Key words are words used to expand search criteria for readers. If the word is in the title, it does not need to be repeated in key words. For example: youth, soccer, etc.

Authors’ response: The key words were now changed to the following:

“Keywords: young; football; imbalance; power training, peak height velocity”

Methods

Lines 126-128: You must have inputted more parameters to calculate the sample size. For example what was the test family, statistical test, project effect size, correlation among measures, number of groups and measurements, etc.? Or was the estimate based on means and SD from prior similar studies? If so provide the references.

Authors’ response: We thank the reviewer for this insightful comment. We now provide detailed information on the parameters used for the a priori sample size calculation. The sample size was estimated using G*Power (version 3.1.9.7) based on a mixed-model ANOVA design (test family: F-tests; statistical test: repeated-measures, within–between interaction). The calculation assumed two groups (prepubertal vs. post-pubertal) and two measurement time points. A medium projected effect size (f = 0.25), an α level of 0.05, a desired power of 0.80, and a correlation among repeated measures of 0.50 were entered. These values were selected based on previously reported effect sizes and variability in youth soccer and maturity-related training studies (Hammami et al., 2020; Moran et al., 2017; Radnor et al., 2018). The analysis indicated a minimum required sample of 52 participants; therefore, 64 players were recruited to accommodate potential dropouts. All details have now been added to the Methods section as follow:

“The sample size was determined a priori using G*Power version 3.1.9.7 (University of Düsseldorf, Germany). The calculation was based on a mixed-model ANOVA (test family: F-tests; statistical test: repeated-measures, within–between interaction), with two groups (prepubertal vs. post-pubertal) and two measurement time points. A projected medium effect size (f = 0.25), an α level of 0.05, a desired statistical power of 0.80, and an assumed correlation among repeated measures of 0.50 were used. These parameters were derived from reported effect sizes and variability in previous youth soccer and maturity-related training interventions (Hammami et al., 2020; Moran et al., 2017; Radnor et al., 2018). The analysis indicated that a minimum of 52 participants was required; therefore, 64 players were recruited to account for potential attrition.”

Line 181: Was the acronym ICC defined previously. If not, then it needs to be defined the first time it appears.

References used: Defined as suggested. Thank you.

Line 185: Have you previously defined how SWC is calculated. If I remember correctly it is the performance SD x the effect size of interest (e.g., small effect size would = 0.2). The reader needs to know how SWC is calculated.

References used: Defined as suggested. Thank you.

Results

Line 196-197: Authors state: “except for inter-limb asymmetry during the re-test, which was borderline significant (Table 1).” What is borderline significant (p=0.06 or p=0.1 or p=0.2???). You refer to Table 1 but there are no p values provided in table 1 so we have no idea what borderline means.

Authors’ responses: Dear Reviewer, we appreciate your insightful remark. In response, we have revised the sentence and added the p-value to Table 1 as suggested.

Table 2 : Please provide all the precise p values not just that all were p<0.05.

Lines 208-210: Provide the range of p values.

Authors’ responses:All details were added as suggested. Thank you

Line 215: Commendable is not a standard description of the strength of the reliability scores. Typically, as you state in the statistical analysis section they are termed as excellent, good, moderate etc.

Authors’ responses: We thank the reviewer for this observation. We agree that “commendable” is not a standard descriptor for reliability outcomes. In accordance with the terminology used in the statistical analysis section, we have replaced “commendable” with standardized reliability classifications (i.e., excellent, good, moderate, or poor) based on the corresponding ICC thresholds. The text has been updated accordingly to ensure consistency and adherence to accepted reporting standards.

Discussion

Well written and organized.

Authors’ responses: We thank the reviewer for the positive feedback and appreciation of the manuscript’s organization and clarity.

Attachment Submitted filename: Point-by-point revisions-R1.pdf

# Peer review: Decision Letter 1

**DOI:** 10.1371/journal.pone.0341344.r003

**Authors:** Holakoo Mohsenifar, Holakoo Mohsenifar

22 Dec 2025

PONE-D-25-55309R1

Inter-limb asymmetry during horizontal jump test in youth soccer: Evaluating reliable and sensitive metrics through maturation

PLOS One

Dear Dr. Hammami,

Thank you for submitting your manuscript to PLOS ONE. After careful consideration, we feel that it has merit but does not fully meet PLOS ONE’s publication criteria as it currently stands. Therefore, we invite you to submit a revised version of the manuscript that addresses the points raised during the review process.

Please submit your revised manuscript by Feb 05 2026 11:59PM. If you will need more time than this to complete your revisions, please reply to this message or contact the journal office at plosone@plos.org. When you're ready to submit your revision, log on to https://www.editorialmanager.com/pone/ and select the 'Submissions Needing Revision' folder to locate your manuscript file.

Please include the following items when submitting your revised manuscript:

- A letter that responds to each point raised by the academic editor and reviewer(s). You should upload this letter as a separate file labeled 'Response to Reviewers'.
- A marked-up copy of your manuscript that highlights changes made to the original version. You should upload this as a separate file labeled 'Revised Manuscript with Track Changes'.
- An unmarked version of your revised paper without tracked changes. You should upload this as a separate file labeled 'Manuscript'.

-->If you would like to make changes to your financial disclosure, please include your updated statement in your cover letter. Guidelines for resubmitting your figure files are available below the reviewer comments at the end of this letter.

If applicable, we recommend that you deposit your laboratory protocols in protocols.io to enhance the reproducibility of your results. Protocols.io assigns your protocol its own identifier (DOI) so that it can be cited independently in the future. For instructions see: https://journals.plos.org/plosone/s/submission-guidelines#loc-laboratory-protocols. Additionally, PLOS ONE offers an option for publishing peer-reviewed Lab Protocol articles, which describe protocols hosted on protocols.io. Read more information on sharing protocols at https://plos.org/protocols?utm_medium=editorial-email&utm_source=authorletters&utm_campaign=protocols.

We look forward to receiving your revised manuscript.

Kind regards,

Holakoo Mohsenifar

Academic Editor

PLOS One

**Journal Requirements:**

1. If the reviewer comments include a recommendation to cite specific previously published works, please review and evaluate these publications to determine whether they are relevant and should be cited. There is no requirement to cite these works unless the editor has indicated otherwise.

2. Please review your reference list to ensure that it is complete and correct. If you have cited papers that have been retracted, please include the rationale for doing so in the manuscript text, or remove these references and replace them with relevant current references. Any changes to the reference list should be mentioned in the rebuttal letter that accompanies your revised manuscript. If you need to cite a retracted article, indicate the article’s retracted status in the References list and also include a citation and full reference for the retraction notice.

[Note: HTML markup is below. Please do not edit.]

Reviewers' comments:

Reviewer's Responses to Questions

**Comments to the Author**

1. If the authors have adequately addressed your comments raised in a previous round of review and you feel that this manuscript is now acceptable for publication, you may indicate that here to bypass the “Comments to the Author” section, enter your conflict of interest statement in the “Confidential to Editor” section, and submit your "Accept" recommendation.

Reviewer #1: All comments have been addressed

Reviewer #2: (No Response)

**********

2. Is the manuscript technically sound, and do the data support the conclusions?

The manuscript must describe a technically sound piece of scientific research with data that supports the conclusions. Experiments must have been conducted rigorously, with appropriate controls, replication, and sample sizes. The conclusions must be drawn appropriately based on the data presented.

Reviewer #1: Yes

Reviewer #2: Yes

**********

3. Has the statistical analysis been performed appropriately and rigorously?

Reviewer #1: Yes

Reviewer #2: Yes

**********

4. Have the authors made all data underlying the findings in their manuscript fully available?

The PLOS Data policy requires authors to make all data underlying the findings described in their manuscript fully available without restriction, with rare exception (please refer to the Data Availability Statement in the manuscript PDF file). The data should be provided as part of the manuscript or its supporting information, or deposited to a public repository. For example, in addition to summary statistics, the data points behind means, medians and variance measures should be available. If there are restrictions on publicly sharing data—e.g. participant privacy or use of data from a third party—those must be specified.

Reviewer #1: Yes

Reviewer #2: Yes

**********

5. Is the manuscript presented in an intelligible fashion and written in standard English?

PLOS ONE does not copyedit accepted manuscripts, so the language in submitted articles must be clear, correct, and unambiguous. Any typographical or grammatical errors should be corrected at revision, so please note any specific errors here.

Reviewer #1: Yes

Reviewer #2: Yes

**********

6. Review Comments to the Author

Please use the space provided to explain your answers to the questions above. You may also include additional comments for the author, including concerns about dual publication, research ethics, or publication ethics. (Please upload your review as an attachment if it exceeds 20,000 characters)

Reviewer #1: Thank you for thoroughly addressing and responding to all the comments. The article is now ready for publication

Reviewer #2: Authors have addressed most of my prior suggeastions. There are a couple of very minor revisions needed:

1. The proper term is prepubertal not prepuberal (add the letter "t'). This misspelling occurs throughout the manuscript.

2. ICC is defined as intraclass correlation coefficient : p. 8 line 189

3. Line 226: Change unveiled to revealed.

**********

7. PLOS authors have the option to publish the peer review history of their article (what does this mean?). If published, this will include your full peer review and any attached files.

If you choose “no”, your identity will remain anonymous but your review may still be made public.

**Do you want your identity to be public for this peer review?**   For information about this choice, including consent withdrawal, please see our Privacy Policy.

Reviewer #1: No

Reviewer #2: **Yes:**   David Behm

**********

[NOTE: If reviewer comments were submitted as an attachment file, they will be attached to this email and accessible via the submission site. Please log into your account, locate the manuscript record, and check for the action link "View Attachments". If this link does not appear, there are no attachment files.]

To ensure your figures meet our technical requirements, please review our figure guidelines: https://journals.plos.org/plosone/s/figures

You may also use PLOS’s free figure tool, NAAS, to help you prepare publication quality figures: https://journals.plos.org/plosone/s/figures#loc-tools-for-figure-preparation.

NAAS will assess whether your figures meet our technical requirements by comparing each figure against our figure specifications.

# Author comment: Author response to Decision Letter 2

**DOI:** 10.1371/journal.pone.0341344.r004

25 Dec 2025

Plos One Manuscript: PONE-D-25-55309

Title: Inter-limb asymmetry during horizontal jump test in youth soccer: Evaluating reliable

and sensitive metrics through maturation

Second revision

Dear Editor,

Dear Reviewers,

We would like to express our gratitude for your valuable time you have invested in our manuscript

and the constructive and helpful comments on the revised paper. We have responded to all of your

queries in point-by-point responses. Whenever needed, we have made changes to the re-revised

manuscript. We hope that you will find the current version of our manuscript suitable for

publication in Plos One.

Kind regards

Prof. Raouf Hammami, PhD On behalf of all co-authors

Reviewer #1

Thank you for thoroughly addressing and responding to all the comments. The article is now ready

for publication.

Authors’ response: We sincerely thank the Editor and Reviewers for their time and valuable

feedback. We have carefully addressed all comments and are grateful for the positive assessment

that the manuscript is now ready for publication.

Reviewer #2: Changes are highlighted in yellow

Authors have addressed most of my prior suggestion’s. There are a couple of very minor revisions

needed:

1. The proper term is prepubertal not prepuberal (add the letter "t'). This misspelling occurs

throughout the manuscript.

Authors’ response: Thank you for pointing this out. The term has been corrected from prepuberal

to prepubertal throughout the entire manuscript.

2. ICC is defined as intraclass correlation coefficient: p. 8 line 189

Authors’ response: We thank the Reviewer for this comment. The definition of ICC has been

included at p. 8, line 189.

3. Line 226: Change unveiled to revealed.

Authors’ response: Thank you for this suggestion. The term unveiled has been corrected to

revealed in the manuscript

Attachment Submitted filename: Point-bypoint revisions-R2.pdf

# Peer review: Decision Letter 2

**DOI:** 10.1371/journal.pone.0341344.r005

**Authors:** Holakoo Mohsenifar, Holakoo Mohsenifar, Stefano Amatori

6 May 2026

PONE-D-25-55309R2

Inter-limb asymmetry during horizontal jump test in youth soccer: Evaluating reliable and sensitive metrics through maturation

PLOS One

Dear Dr. Hammami,

Thank you for submitting your manuscript to PLOS ONE. After careful consideration, we feel that it has merit but does not fully meet PLOS ONE’s publication criteria as it currently stands. Therefore, we invite you to submit a revised version of the manuscript that addresses the points raised during the review process.

Please submit your revised manuscript by Jun 20 2026 11:59PM. If you will need more time than this to complete your revisions, please reply to this message or contact the journal office at plosone@plos.org. When you're ready to submit your revision, log on to https://www.editorialmanager.com/pone/ and select the 'Submissions Needing Revision' folder to locate your manuscript file.

Please include the following items when submitting your revised manuscript:

- A letter that responds to each point raised by the academic editor and reviewer(s). You should upload this letter as a separate file labeled 'Response to Reviewers'.
- A marked-up copy of your manuscript that highlights changes made to the original version. You should upload this as a separate file labeled 'Revised Manuscript with Track Changes'.
- An unmarked version of your revised paper without tracked changes. You should upload this as a separate file labeled 'Manuscript'.

If you would like to make changes to your financial disclosure, please include your updated statement in your cover letter. Guidelines for resubmitting your figure files are available below the reviewer comments at the end of this letter.

If applicable, we recommend that you deposit your laboratory protocols in protocols.io to enhance the reproducibility of your results. Protocols.io assigns your protocol its own identifier (DOI) so that it can be cited independently in the future. For instructions see: https://journals.plos.org/plosone/s/submission-guidelines#loc-laboratory-protocols. Additionally, PLOS ONE offers an option for publishing peer-reviewed Lab Protocol articles, which describe protocols hosted on protocols.io. Read more information on sharing protocols at https://plos.org/protocols?utm_medium=editorial-email&utm_source=authorletters&utm_campaign=protocols.

As the corresponding author, your ORCID iD is verified in the submission system and will appear in the published article. PLOS supports the use of ORCID, and we encourage all coauthors to register for an ORCID iD and use it as well. Please encourage your coauthors to verify their ORCID iD within the submission system before final acceptance, as unverified ORCID iDs will not appear in the published article. *Only*   the individual author can complete the verification step; PLOS staff *cannot*   verify ORCID iDs on behalf of authors.

We look forward to receiving your revised manuscript.

Kind regards,

Stefano Amatori, Ph.D.

Academic Editor

PLOS One

**Journal Requirements:**

If the reviewer comments include a recommendation to cite specific previously published works, please review and evaluate these publications to determine whether they are relevant and should be cited. There is no requirement to cite these works unless the editor has indicated otherwise.

[Note: HTML markup is below. Please do not edit.]

Reviewers' comments:

Reviewer's Responses to Questions

**Comments to the Author**

1. If the authors have adequately addressed your comments raised in a previous round of review and you feel that this manuscript is now acceptable for publication, you may indicate that here to bypass the “Comments to the Author” section, enter your conflict of interest statement in the “Confidential to Editor” section, and submit your "Accept" recommendation.

Reviewer #1: All comments have been addressed

Reviewer #2: All comments have been addressed

Reviewer #3: (No Response)

**********

2. Is the manuscript technically sound, and do the data support the conclusions?

The manuscript must describe a technically sound piece of scientific research with data that supports the conclusions. Experiments must have been conducted rigorously, with appropriate controls, replication, and sample sizes. The conclusions must be drawn appropriately based on the data presented.

Reviewer #1: Yes

Reviewer #2: Yes

Reviewer #3: Yes

**********

3. Has the statistical analysis been performed appropriately and rigorously?

Reviewer #1: Yes

Reviewer #2: Yes

Reviewer #3: Yes

**********

4. Have the authors made all data underlying the findings in their manuscript fully available?

The PLOS Data policy requires authors to make all data underlying the findings described in their manuscript fully available without restriction, with rare exception (please refer to the Data Availability Statement in the manuscript PDF file). The data should be provided as part of the manuscript or its supporting information, or deposited to a public repository. For example, in addition to summary statistics, the data points behind means, medians and variance measures should be available. If there are restrictions on publicly sharing data—e.g. participant privacy or use of data from a third party—those must be specified.

Reviewer #1: Yes

Reviewer #2: Yes

Reviewer #3: Yes

**********

5. Is the manuscript presented in an intelligible fashion and written in standard English?

PLOS ONE does not copyedit accepted manuscripts, so the language in submitted articles must be clear, correct, and unambiguous. Any typographical or grammatical errors should be corrected at revision, so please note any specific errors here.

Reviewer #1: Yes

Reviewer #2: Yes

Reviewer #3: Yes

**********

6. Review Comments to the Author

Please use the space provided to explain your answers to the questions above. You may also include additional comments for the author, including concerns about dual publication, research ethics, or publication ethics. (Please upload your review as an attachment if it exceeds 20,000 characters)

Reviewer #1: I would like to congratulate the authors for their careful revision of the manuscript. They have addressed my comments and recommendations appropriately, and the responses provided are clear and satisfactory. The changes made have improved the quality and clarity of the paper. Thank you for your efforts in revising the manuscript.

Reviewer #2: Authors have addressed my second round of requested revisions.

XXXXXXXXXXXXXXXXXXXXXXXXXXXXXXXXXXXXX

Reviewer #3: I would like to thank the authors for the opportunity to review this manuscript. The study addresses a relevant and timely question in youth soccer research, applying a sound test–retest design to examine the reliability and sensitivity of inter-limb asymmetry across maturity stages. The clear stratification by APHV, the use of established reliability and sensitivity metrics (ICC, SEM, SWC), and the practical orientation of the work are notable strengths. The points below are intended as constructive suggestions to further strengthen the methodological transparency and the interpretive precision of the manuscript.

General comment

The title and several passages in the Abstract and Discussion frame inter-limb asymmetry as an indicator for "prescribing power training priorities" and for "designing targeted training interventions." However, the cross-sectional design — without injury or longitudinal performance outcomes — does not support inferences about training prescription, transfer effects, or injury-related implications. In line with recent commentary on this issue (Afonso et al., 2026), I recommend aligning the title and conclusions with what the data actually address: test–retest reliability and sensitivity of the SLHT-derived asymmetry score across two maturity groups.

Participant classification

The competitive level of the players is not specified. I recommend classifying the sample according to McKay et al. (2022), ideally applying the scoring criteria operationalised by Wilkins et al. (2025) to support the reliability of that classification and the generalisability of the findings.

Order of limb testing

It is not stated whether the order of testing between the dominant and non-dominant limb was randomised, counterbalanced, or fixed. Given that the asymmetry score is computed directly from the between-limb difference, this detail should be clarified.

Movement goal and attentional focus

The authors report that participants were instructed to "execute a maximal forward hop," without further detail on verbal instructions, movement goal, or attentional focus. Evidence indicates that these factors meaningfully affect lower-limb ballistic performance and its variability (Pompa et al., 2026), with direct implications for the SEM and SWC values reported here. Please specify the exact instructions used, or acknowledge this among the limitations.

Swing leg and propulsion strategy

It is not clarified whether participants were allowed a swing-leg drive or whether this was constrained. An unrestricted swing-leg action increases forward momentum and may differentially affect dominant vs. non-dominant performance, particularly in younger players. Please specify the procedure.

Asymmetry vs. coordinative differences in younger players

The Discussion states that "asymmetry appeared more pronounced in younger players, consistent with previous findings that asymmetries decline with maturation." The greater between-limb difference observed in the prepubertal group may, however, reflect inter-limb coordinative variability and lower motor-control stability rather than a true neuromuscular asymmetry. This distinction carries different practical implications, and recent work has discussed how dominant-limb preference, repetitive sport-specific gestures, and developmental coordination interact in young soccer players (D'Onofrio et al., 2025). I recommend reframing this passage accordingly.

Stretch-shortening cycle and citation support

The statement that "the hop test heavily recruits the stretch-shortening cycle, which enhances elastic energy storage and release, allowing for more forceful concentric actions [35–38]" is not substantively supported by the cited references. Hammami et al. (2023) discuss SSC efficiency generically as a training adaptation, without characterising the SLHT as an SSC-dependent task; Andrade et al. (2020) examined bilateral drop jumps in volleyball players, with only speculative reference to elastic energy; Radnor et al. (2020) addresses muscle architecture across maturity, not SSC mechanics in hop tests; and Taube et al. (2012) investigated neuromuscular adaptations to drop jump training, focusing on reflex contributions and joint stiffness. I recommend either reformulating the statement to match what the cited sources report, or replacing the citations with primary sources specifically characterising the biomechanics of single-leg hop tests.

Suggested references

Afonso J, Andrade R, Oliveira E, Turner A, Warneke K, Bishop C. Asymmetries and injury risk: when titles spin the tale. J Strength Cond Res. 2026;40(5):e530–e533. doi:10.1519/JSC.0000000000005430

D'Onofrio R, Perna P, Pompa D, Civitillo C, Sannicandro I, Manzi V. Asymmetry, lumbo-pelvic hip complex and injury in European soccer players. J Sports Med Phys Fitness. 2025;65(0):000–000. doi:10.23736/S0022-4707.25.16845-X

McKay AKA, Stellingwerff T, Smith ES, et al. Defining training and performance caliber: a participant classification framework. Int J Sports Physiol Perform. 2022;17(2):317–331.

Wilkins L, et al. Reliability of participant classification in sport and exercise science: application of McKay et al.'s (2022) framework. J Sports Sci. 2025;43:2914–2926.

Pompa D, Carson HJ, Romagnoli R, et al. Effect of movement goal on countermovement jump performance in athletes across different sports. Sci Rep. 2026;16:9748. doi:10.1038/s41598-026-40260-9

**********

7. PLOS authors have the option to publish the peer review history of their article (what does this mean?). If published, this will include your full peer review and any attached files.

If you choose “no”, your identity will remain anonymous but your review may still be made public.

**Do you want your identity to be public for this peer review?**   For information about this choice, including consent withdrawal, please see our Privacy Policy.

Reviewer #1: No

Reviewer #2: **Yes:**   David Behm

Reviewer #3: No

**********

[NOTE: If reviewer comments were submitted as an attachment file, they will be attached to this email and accessible via the submission site. Please log into your account, locate the manuscript record, and check for the action link "View Attachments". If this link does not appear, there are no attachment files.]

To ensure your figures meet our technical requirements, please review our figure guidelines: https://journals.plos.org/plosone/s/figures

You may also use PLOS’s free figure tool, NAAS, to help you prepare publication quality figures: https://journals.plos.org/plosone/s/figures#loc-tools-for-figure-preparation.

NAAS will assess whether your figures meet our technical requirements by comparing each figure against our figure specifications.

# Author comment: Author response to Decision Letter 3

**DOI:** 10.1371/journal.pone.0341344.r006

1 Jun 2026

Plos One

Manuscript: PONE-D-25-55309

Title: Test–Retest Reliability and Sensitivity of Horizontal Jump Inter-Limb Asymmetry in Youth Soccer Players Across Maturity Stages

Third revision

Dear Editor,

Dear Reviewers,

We would like to express our gratitude once again for your valuable time you have invested in our manuscript and the constructive and helpful comments on the revised paper. We have responded to all of your queries in point-by-point responses. Whenever needed, we have made changes to the re-revised manuscript. We hope that you will find the current version of our manuscript suitable for publication in Plos One.

Kind regards

Prof. Raouf Hammami, PhD On behalf of all co-authors

Reviewer #1: I would like to congratulate the authors for their careful revision of the manuscript. They have addressed my comments and recommendations appropriately, and the responses provided are clear and satisfactory. The changes made have improved the quality and clarity of the paper. Thank you for your efforts in revising the manuscript.

Authors’ response: We sincerely thank the reviewer for the positive evaluation of our revised manuscript. We greatly appreciate your careful assessment and encouraging comments. We are pleased that our revisions and responses have satisfactorily addressed your concerns and that the changes made have improved the quality and clarity of the paper. Your valuable feedback has been highly helpful in strengthening the manuscript. Thank you again for your time and consideration.

Reviewer #2: Authors have addressed my second round of requested revisions.

Authors’ response: We sincerely thank the reviewer for the careful evaluation of our revised manuscript. We appreciate your recognition that the requested second-round revisions have been adequately addressed. Your constructive feedback has been invaluable in improving the quality of our work. Thank you again for your time and consideration.

Reviewer #3: Changes are highlighted in yellow

I would like to thank the authors for the opportunity to review this manuscript. The study addresses a relevant and timely question in youth soccer research, applying a sound test–retest design to examine the reliability and sensitivity of inter-limb asymmetry across maturity stages. The clear stratification by APHV, the use of established reliability and sensitivity metrics (ICC, SEM, SWC), and the practical orientation of the work are notable strengths. The points below are intended as constructive suggestions to further strengthen the methodological transparency and the interpretive precision of the manuscript.

Authors’ response: We sincerely thank the reviewer for the thorough and thoughtful evaluation of our manuscript. We greatly appreciate your positive comments regarding the relevance and timeliness of the study, as well as your recognition of the methodological strengths of our work, including the test–retest design, the stratification by APHV, and the use of established reliability and sensitivity metrics (ICC, SEM, and SWC). We are also grateful for your acknowledgment of the practical relevance of the study for youth soccer research and practice.

We appreciate the constructive suggestions provided, which have helped us improve the methodological transparency and interpretive precision of the manuscript. All comments have been carefully considered and addressed in the revised version, and we believe that these revisions have further strengthened the quality and clarity of the paper. Thank you again for your valuable feedback and time devoted to reviewing our work.

General comment

The title and several passages in the Abstract and Discussion frame inter-limb asymmetry as an indicator for "prescribing power training priorities" and for "designing targeted training interventions." However, the cross-sectional design — without injury or longitudinal performance outcomes — does not support inferences about training prescription, transfer effects, or injury-related implications. In line with recent commentary on this issue (Afonso et al., 2026), I recommend aligning the title and conclusions with what the data actually address: test–retest reliability and sensitivity of the SLHT-derived asymmetry score across two maturity groups.

Authors’ response: We thank the reviewer for this important and insightful comment. In accordance with your recommendation and the recent commentary by Afonso et al. (2026), we revised the title, Abstract, Discussion, and Conclusion to better align with the scope of the study and the cross-sectional nature of the design. Specifically, we removed statements implying training prescription, transfer effects, or injury-related implications and focused the interpretation on the test–retest reliability and sensitivity of the SLHT-derived inter-limb asymmetry scores across maturity groups.

Participant classification

The competitive level of the players is not specified. I recommend classifying the sample according to McKay et al. (2022), ideally applying the scoring criteria operationalised by Wilkins et al. (2025) to support the reliability of that classification and the generalisability of the findings.

Authors’ response: We thank the reviewer for this valuable suggestion. In the revised manuscript, we have now clarified the competitive level of the participants and classified the sample in accordance with the framework proposed by McKay et al. (2022). In addition, we applied the operationalised scoring criteria outlined by Wilkins et al. (2025) to ensure a more transparent and standardized classification of player level. These additions have been included in the Methods section to improve clarity, reproducibility, and the generalisability of the findings.

Order of limb testing

It is not stated whether the order of testing between the dominant and non-dominant limb was randomised, counterbalanced, or fixed. Given that the asymmetry score is computed directly from the between-limb difference, this detail should be clarified.

Authors’ response: We thank the reviewer for this important point. The order of testing between limbs was kept fixed and identical for all participants and both testing sessions to ensure standardisation of measurement procedures. This clarification has been added to the Methods section.

Movement goal and attentional focus

The authors report that participants were instructed to "execute a maximal forward hop," without further detail on verbal instructions, movement goal, or attentional focus. Evidence indicates that these factors meaningfully affect lower-limb ballistic performance and its variability (Pompa et al., 2026), with direct implications for the SEM and SWC values reported here. Please specify the exact instructions used, or acknowledge this among the limitations.

Authors’ response: We thank the reviewer for this valuable observation. In the study limitation, we have clarified the standardized verbal instructions provided during the single-leg hop test, including the requirement to perform a maximal forward hop while maintaining balance upon landing. We also acknowledge that factors such as verbal cueing, movement goal, and attentional focus may influence ballistic performance variability and, consequently, the SEM and SWC outcomes, as highlighted by Pompa et al. (2026). This consideration has now been included in the limitations section.

Swing leg and propulsion strategy

It is not clarified whether participants were allowed a swing-leg drive or whether this was constrained. An unrestricted swing-leg action increases forward momentum and may differentially affect dominant vs. non-dominant performance, particularly in younger players. Please specify the procedure.

Authors’ response: We thank the reviewer for this important comment. We have now clarified the propulsion strategy used during the single-leg hop test. Specifically, participants kept their hands on their hips throughout the movement, while a natural swing-leg action was permitted to allow coordinated execution of the hop. This information has been added to the Methods section.

Asymmetry vs. coordinative differences in younger players

The Discussion states that "asymmetry appeared more pronounced in younger players, consistent with previous findings that asymmetries decline with maturation." The greater between-limb difference observed in the prepubertal group may, however, reflect inter-limb coordinative variability and lower motor-control stability rather than a true neuromuscular asymmetry. This distinction carries different practical implications, and recent work has discussed how dominant-limb preference, repetitive sport-specific gestures, and developmental coordination interact in young soccer players (D'Onofrio et al., 2025). I recommend reframing this passage accordingly.

Authors’ response: We thank the reviewer for this insightful comment. In response, we revised the Discussion to avoid interpreting greater between-limb differences in younger players solely as neuromuscular asymmetry. Instead, we now acknowledge that these findings may also reflect increased coordinative variability and reduced motor-control stability during early development, consistent with the perspective proposed by D’Onofrio et al. (2025).

Stretch-shortening cycle and citation support

The statement that "the hop test heavily recruits the stretch-shortening cycle, which enhances elastic energy storage and release, allowing for more forceful concentric actions [35–38]" is not substantively supported by the cited references. Hammami et al. (2023) discuss SSC efficiency generically as a training adaptation, without characterising the SLHT as an SSC-dependent task; Andrade et al. (2020) examined bilateral drop jumps in volleyball players, with only speculative reference to elastic energy; Radnor et al. (2020) addresses muscle architecture across maturity, not SSC mechanics in hop tests; and Taube et al. (2012) investigated neuromuscular adaptations to drop jump training, focusing on reflex contributions and joint stiffness. I recommend either reformulating the statement to match what the cited sources report, or replacing the citations with primary sources specifically characterising the biomechanics of single-leg hop tests.

Authors’ response: We thank the reviewer for this suggestion. We have revised the statement to better align with the literature and have supported it with appropriate references, which describe the neuromuscular demands of unilateral hop-based tasks in terms of coordinated force production, dynamic balance, and neuromuscular control during propulsion and landing phases.

Suggested references

Afonso J, Andrade R, Oliveira E, Turner A, Warneke K, Bishop C. Asymmetries and injury risk: when titles spin the tale. J Strength Cond Res. 2026;40(5):e530–e533. doi:10.1519/JSC.0000000000005430

D'Onofrio R, Perna P, Pompa D, Civitillo C, Sannicandro I, Manzi V. Asymmetry, lumbo-pelvic hip complex and injury in European soccer players. J Sports Med Phys Fitness. 2025;65(0):000–000. doi:10.23736/S0022-4707.25.16845-X

McKay AKA, Stellingwerff T, Smith ES, et al. Defining training and performance caliber: a participant classification framework. Int J Sports Physiol Perform. 2022;17(2):317–331.

Wilkins L, et al. Reliability of participant classification in sport and exercise science: application of McKay et al.'s (2022) framework. J Sports Sci. 2025;43:2914–2926.

Pompa D, Carson HJ, Romagnoli R, et al. Effect of movement goal on countermovement jump performance in athletes across different sports. Sci Rep. 2026;16:9748. doi:10.1038/s41598-026-40260-9

Attachment Submitted filename: Response R3-Plos One.pdf

# Peer review: Decision Letter 3

**DOI:** 10.1371/journal.pone.0341344.r007

**Authors:** Holakoo Mohsenifar, Holakoo Mohsenifar, Stefano Amatori, Stefano Amatori

4 Jun 2026

Test–retest reliability and sensitivity of horizontal jump inter-limb asymmetry in youth soccer players across maturity stages

PONE-D-25-55309R3

Dear Dr. Hammami,

We’re pleased to inform you that your manuscript has been judged scientifically suitable for publication and will be formally accepted for publication once it meets all outstanding technical requirements.

Within one week, you’ll receive an e-mail detailing the required amendments. When these have been addressed, you’ll receive a formal acceptance letter and your manuscript will be scheduled for publication.

An invoice will be generated when your article is formally accepted. Please note, if your institution has a publishing partnership with PLOS and your article meets the relevant criteria, all or part of your publication costs will be covered. Please make sure your user information is up-to-date by logging into Editorial Manager at Editorial Manager® and clicking the ‘Update My Information' link at the top of the page. For questions related to billing, please contact billing support.

If your institution or institutions have a press office, please notify them about your upcoming paper to help maximize its impact. If they’ll be preparing press materials, please inform our press team as soon as possible -- no later than 48 hours after receiving the formal acceptance. Your manuscript will remain under strict press embargo until 2 pm Eastern Time on the date of publication. For more information, please contact onepress@plos.org.

Kind regards,

Stefano Amatori, Ph.D.

Academic Editor

PLOS One

Additional Editor Comments (optional):

Reviewers' comments:

Reviewer's Responses to Questions

**Comments to the Author**

1. If the authors have adequately addressed your comments raised in a previous round of review and you feel that this manuscript is now acceptable for publication, you may indicate that here to bypass the “Comments to the Author” section, enter your conflict of interest statement in the “Confidential to Editor” section, and submit your "Accept" recommendation.

Reviewer #3: All comments have been addressed

**********

2. Is the manuscript technically sound, and do the data support the conclusions?

The manuscript must describe a technically sound piece of scientific research with data that supports the conclusions. Experiments must have been conducted rigorously, with appropriate controls, replication, and sample sizes. The conclusions must be drawn appropriately based on the data presented.

Reviewer #3: (No Response)

**********

3. Has the statistical analysis been performed appropriately and rigorously?

Reviewer #3: Yes

**********

4. Have the authors made all data underlying the findings in their manuscript fully available?

The PLOS Data policy requires authors to make all data underlying the findings described in their manuscript fully available without restriction, with rare exception (please refer to the Data Availability Statement in the manuscript PDF file). The data should be provided as part of the manuscript or its supporting information, or deposited to a public repository. For example, in addition to summary statistics, the data points behind means, medians and variance measures should be available. If there are restrictions on publicly sharing data—e.g. participant privacy or use of data from a third party—those must be specified.

Reviewer #3: Yes

**********

5. Is the manuscript presented in an intelligible fashion and written in standard English?

PLOS ONE does not copyedit accepted manuscripts, so the language in submitted articles must be clear, correct, and unambiguous. Any typographical or grammatical errors should be corrected at revision, so please note any specific errors here.

Reviewer #3: Yes

**********

6. Review Comments to the Author

Please use the space provided to explain your answers to the questions above. You may also include additional comments for the author, including concerns about dual publication, research ethics, or publication ethics. (Please upload your review as an attachment if it exceeds 20,000 characters)

Reviewer #3: I appreciate the effort the authors made to respond to my comments, and I believe that the way they addressed them strengthens the manuscript. From my side, the manuscript is now acceptable for publication in its current form

**********

7. PLOS authors have the option to publish the peer review history of their article (what does this mean?). If published, this will include your full peer review and any attached files.

If you choose “no”, your identity will remain anonymous but your review may still be made public.

**Do you want your identity to be public for this peer review?**   For information about this choice, including consent withdrawal, please see our Privacy Policy.

Reviewer #3: **Yes:**   Dario Pompa

**********

# Editor report: Acceptance letter

**DOI:** 10.1371/journal.pone.0341344.r008

**Authors:** Holakoo Mohsenifar, Holakoo Mohsenifar, Stefano Amatori, Stefano Amatori

PONE-D-25-55309R3

PLOS One

Dear Dr. Hammami,

I'm pleased to inform you that your manuscript has been deemed suitable for publication in PLOS One. Congratulations! Your manuscript is now being handed over to our production team.

At this stage, our production department will prepare your paper for publication. This includes ensuring the following:

* All references, tables, and figures are properly cited

* All relevant supporting information is included in the manuscript submission,

* There are no issues that prevent the paper from being properly typeset

You will receive further instructions from the production team, including instructions on how to review your proof when it is ready. Please keep in mind that we are working through a large volume of accepted articles, so please give us a few days to review your paper and let you know the next and final steps.

Lastly, if your institution or institutions have a press office, please let them know about your upcoming paper now to help maximize its impact. If they'll be preparing press materials, please inform our press team within the next 48 hours. Your manuscript will remain under strict press embargo until 2 pm Eastern Time on the date of publication. For more information, please contact onepress@plos.org.

You will receive an invoice from PLOS for your publication fee after your manuscript has reached the completed accept phase. If you receive an email requesting payment before acceptance or for any other service, this may be a phishing scheme. Learn how to identify phishing emails and protect your accounts at https://explore.plos.org/phishing.

If we can help with anything else, please email us at customercare@plos.org.

Thank you for submitting your work to PLOS ONE and supporting open access.

Kind regards,

PLOS ONE Editorial Office Staff

on behalf of

Prof. Stefano Amatori

Academic Editor

PLOS One
